# Optimal medical therapy in patients with reduced left ejection fraction undergoing coronary artery bypass graft surgery

**DOI:** 10.1186/s12872-025-05384-2

**Published:** 2025-11-27

**Authors:** Anna Jonsson Holmdahl, Saman Sharif, Erik Birring, Mattias Karlsson, Krister Lindmark

**Affiliations:** 1 Department of Public Health and Clinical Medicine, Umeå University, Umeå, SE 90185 Sweden; 2 Department of Clinical Sciences, Cardiology, Danderyd Hospital, Karolinska Institutet, Stockholm, Sweden

**Keywords:** Heart failure, Secondary prevention, Optimal medical therapy, Coronary artery bypass grafting

## Abstract

**Background:**

Our aim was to explore clinical outcomes between patients with- and without of optimal medical therapy (OMT) in patients with reduced left ventricular ejection fraction (LVEF) undergoing coronary artery bypass graft surgery (CABG).

**Methods:**

In this single-centre observational study, all patients who underwent isolated CABG with a preoperative LVEF of ≤ 40% between 2012 and 2021 were included. Individual data were collected from the medical health records and the register Carath. OMT was defined as prescription of renin-angiotensin-aldosterone system inhibitor (RAAS-I), beta-blocker (BB), Acetylsalicylic acid (ASA) and statins.

**Results:**

102 patients were identified. The frequency of patients with OMT was 44% at admission and 67% 72%, 69% and 68% at discharge, 2 months, 1 year and 2 years follow-up. Patients with OMT at discharge had a significant longer median time to the composite endpoint of first-time hospitalization and all-cause mortality compared to the No-OMT group (1.7 versus 7.1 years, log rank *p* = 0.0038). OMT at discharge was associated with a lower adjusted risk of all-cause mortality and first-time hospitalization for heart failure (adjusted hazard ratio, 0.25; 95% confidence interval, 0.11–0.58; p = < 0.001).

**Conclusions:**

The prescription rate of OMT increased until the 2 months follow-up with no further increases in dose at 1 year and 2 years follow-up. Patients undergoing CABG with a preoperative reduced left ventricular ejection fraction that were prescribed OMT at discharge had better outcomes in terms of a reduced risk of hospitalization for heart failure and all-cause mortality.

## Background

The most common cause of heart failure is coronary artery disease (CAD) [1]. Patients with heart failure with reduced ejection fraction (HFrEF) have a twofold risk for mortality and rehospitalization for heart failure after coronary artery bypass grafting (CABG) compared to patients without heart failure [2]. Even with this higher risk, the Surgical Treatment for Ischemic Heart Failure (STICH) trial showed long-term survival benefits in patients with HFrEF and CAD undergoing CABG compared to medical therapy alone [3, 4]. Several studies have showed the importance of adherence to optimal medical therapy in patients with HFrEF [5]. Early initiation and up-titration of heart failure medical therapy in patients with acute heart failure has proven better clinical outcomes within the first 180 days post-discharge but few studies on medical treatment for heart failure patients with HFrEF undergoing CABG have been conducted [6, 7].

There are several potential reasons for not initiating or up titrating heart failure medications after a cardiac surgery. Patients with preoperatively left ventricular function (LVEF) under 40% has a markedly increased risk of low cardiac output syndrome after isolated CABG [8]. Further, renal failure is twice as common in patients with HFrEF undergoing CABG compared to patients with normal ejection fraction [9]. Hence, the number of eligible patients without contraindications to medical heart failure treatment are uncertain. We sought to evaluate the prescription rate, intensity and effect of optimal medical treatment including heart failure specific drugs in a ”real world” population of patients with HFrEF undergoing CABG at a tertiary care university centre.

## Materials and methods

### Study population

All patients with a preoperative LVEF of ≤ 40% who underwent an isolated CABG surgery with between the 1 st of August 2012 to the 1 st of august 2021 and were residents in Västerbotten county were included in this study. Exclusion criteria were concomitant valve- or aortic surgery.

Patients were identified from the quality register Carath. In Carath all patients that underwent cardiac surgery at the thoracic surgery department in Umeå University Hospital have been registered since 2005. Variables from Carath include several pre-, intra- and postoperative parameters. All patients meeting the inclusion criteria were identified in Carath. Additional data on medical therapy and echocardiogram parameters were collected from the electronical health records (EHR) at admission, discharge and 2 months, 1 year and 2 years post-surgery as well as highest post operative serum-creatinine and serum-potassium during hospitalization. The date for first time hospitalization due to heart failure and death were collected from the EHR during the total follow-up time after surgery. Optimal medical therapy (OMT) was defined as prescription of renin-angiotensin-aldosterone system inhibitor (RAAS-I), betablocker (BB), statins and Acetyl Salicylic Acid (ASA). The percentage of target dose for each heart failure medication were calculated using the recommendations from European Society of Cardiology (ESC) [10]. Target-dose OMT was defined as at least 50% of target dose for RAAS-I (including angiotensin-converting-enzyme inhibitor (ACE-I), angiotensin II receptor blocker (ARB) or angiotensin receptor/neprilysin inhibitor (ARNI)), and BB in addition of ASA and statins. Since this was an observational study, we did not follow a specified protocol for OMT at discharge and was left to the discretion of the responsible surgeon. The patients later had a routine follow-up visit with a cardiologist at two months post-surgery and then had follow-ups with a primary care physician in most of the cases. Estimated glomerular filtration rate (eGFR) was calculated by the revised Lund-Malmo GFR estimating equation [11].

### Statistical analysis

Categorical variables are presented with frequencies and percentage and analysed with Pearson’s Chi-squared test. Continuous variables without normal distribution are presented as medians with interquartile range and analysed with Mann-Whitney U Test. Continuous variables are presented as means with standard deviations and analysed with Student’s t-test. Paired parametric variables were analysed with paired t-test and categorical paired variables were analysed with McNemar test.

The effect of optimal medical therapy on the combined outcome of all-cause death and first-time heart failure hospitalization was analysed using the Kaplan-Meier method and compared using the log-rank test and with the Cox proportional hazard (PH) regression model. The Kaplan-Meier curves were truncated when the number at risk dropped to less than 10 or < 10% of the initial population at risk. The aim with the analysis was to create a causal model with the aim to evaluate the effect of OMT on the endpoints. Hence, all covariates in the analysis represents potential differences between the exposure groups that could affect the outcome. We performed the Cox PH regression analysis adjusting for the following covariates at admission: age, sex, body mass index, diabetes mellitus, hypertension, hyperlipidaemia, previous stroke, heart failure diagnosis, previous myocardial infarction, chronic obstructive pulmonary disease, peripheral artery disease, acute coronary syndrome as indication for CABG (unstable angina or recent myocardial infarction), left ventricular ejection fraction, eGFR, pulmonal hypertension, stroke after surgery, atrial fibrillation after surgery. Assumptions of proportionality of hazard were verified by log-log plots.

SPSS Statistics (29.0.1.0) was used for statistical analysis. A p value less than 0.05 was considered statistically significant.

## Results

### Study population

**Fig. 1:**
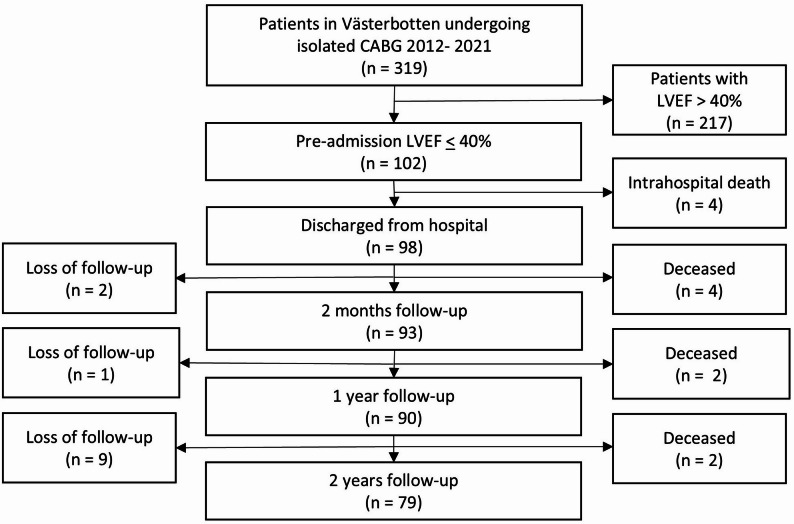
Flowchart of patient selection

A total of 102 patients were included in the study (Fig. 1). As seen in Fig. 1, the study population consisted of 32% of the CABG patients. During the first two years of follow-up time 12 patients were lost to follow-up. The median follow-up time was 3.6 years (IQR 2.7–6.0.7.0 years). During follow-up time 26 patients (26%) died, and 25 patients (25%) were hospitalized due to heart failure.

At admission there were only 13% women, and the mean age was 67 years (Table 1). There was a high burden of comorbidities, with hypertension and hyperlipemia being the most common.

**Table 1:** Patient characteristics by prescription of target-dose OMT at discharge

Characteristics	All patients at admission (*n* = 102)	Target-dose OMT *n* = 22)	No target-dose OMT (*n* = 76)	*p*
Women - n (%)	13 (13)	2 (9)	10 (13)	1.000
Age - years, mean (SD)	67 (9)	64 (9)	67 (9)	0.137
*≥* 75 years	24 (24)	3 (14)	19 (25)	0.386
BMI - kg/m2 median (IQR)	27 (4)	28 (26–31)	27 (24–29)	0.067
NYHA III-IV - n (%)	71 (70)	16 (73)	52 (68)	0.700
EuroScore II (%), median (IQR)	2 (2–4)	2,6 (1,6 − 3,7)	2,2 (1,6 − 3,8)	0.838
eGFR category (ml/min/1.73 m2)				
*≥* 90	5 (5)	2 (9)	3 (4)	0.312
60-<90	78 (77)	15 (68)	62 (82)	0.237
30-<60	18 (18)	5 (23)	10 (13)	0.316
<30	1 (1)	0	1 (1)	1.000
Haemoglobin (g/L) mean (SD)	140 (16)	138 (16)	140 (16)	0.585
Left ventricular ejection fraction				0.524
30–40%	84 (83)	17 (77)	64 (84)	
<30%	17 (17)	5 (23)	12 (16)	
Medical history - n (%)				
Smoking	57 (56)	9 (41)	48 (63)	0.062
Diabetes	33 (32)	10 (46)	22 (29)	0.146
Prior PCI intervention	18 (18)	2 (9)	16 (21)	0.202
COPD	3 (3)	0	3 (4)	1.000
Cerebrovascular disease	15 (15)	3 (14)	12 (16)	1.000
Non-cardiac vascular disease	8 (8)	2 (9)	6 (8)	1.000
Hypertension	80 (80)	20 (91)	60 (79)	0.348
Hyperlipidaemia	78 (77)	19 (86)	56 (74)	0.217
Previous heart failure diagnosis	70 (69)	17 (77)	50 (66)	0.308
Atrial fibrillation/atrial flutter	11 (11)	5 (23)	6 (8)	0.117
Previous heart surgery	2 (2)	1 (4,5)	1 (1)	0.400
Indication for surgery - n (%)				
Instable angina	46 (45)	6 (27)	37 (49)	0.075
Recent myocardial infarction (within 3 w)	53 (52)	8 (36)	39 (51)	0.335
Emergency or Urgent CABG surgery	66 (65)	10 (46)	53 (70)	0.036
Inotropes	12 (12)	3 (14)	7 (9)	0.689
Severity of CAD – n (%)				
SVD	1 (1)	0 (0)	1 (1)	1.000
MVD	101 (99)	22 (100)	75 (99)	1.000
LM	26 (25)	2 (9)	23 (30)	0.045
Medical treatment at admission – n (%)				
Statins	69 (68)	22 (100)	60 (79)	0.019
ASA	64 (63)	22 (100)	65 (86)	0.066
Betablocker *≥* 50% of target dose	42 (41)	22 (100)	20 (26)	< 0.001
RAAS inhibitor > 50% of target dose	53 (52)	22 (100)	31 (41)	< 0.001
MRA > 50% of target dose	38 (37)	15 (68)	23 (30)	0.001
Anticoagulants	9 (9)	4 (18)	5 (7)	0.202
Post-operative complications – n (%)				
Atrial fibrillation/atrial flutter at discharge – n (%)	20 (20)	4 (18)	15 (20)	1.000
Postoperative pneumonia – n (%)	4 (4)	2 (9)	2 (3)	0.217
Permanent pacemaker - n (%)	3 (3)	0	3 (4)	1.000
Pleural effusion drainage - n (%)	3 (3)	0	3 (4)	1.000
Stroke - n (%)	3(3)	2 (9)	0	0.049
Reoperation for bleeding - (%)	10 (10)	3 (14)	5 (7)	0.373
Postoperative p-creatinine value over threshold of 221 µmol/L – n (%)	5 (5)	1 (1)	4 (5)	1.000
Postoperative s-potassium value over threshold of 5.0 mmol/L – n (%)	17 (17)	6 (27)	11 (15)	0.205

*BMI* Body mass index, *NYHA* New York Heart Association Functional Classification, *COPD* chronic

obstructive pulmonary disease, *PCI* Percutaneous coronary intervention, *CABG* coronary artery bypass graft

surgery, *ASA* Acetylsalicylic Acid, *RAAS* renin–angiotensin–aldosterone system, *MRA* mineralocorticoid

receptor antagonist, *OMT* optimal medical therapy, *CAD* coronary artery disease

### Prescription of optimal medical therapy

**Fig. 2:**
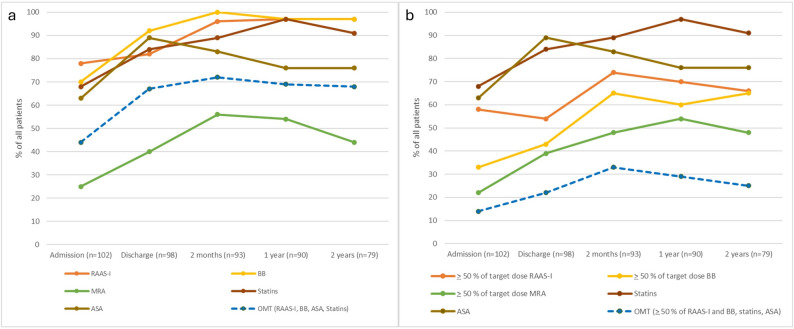
(**a**) Prescription rate of OMT. (**b**) Prescription rate of OMT defined as *≥* 50% of target doses for RAAS-I and BB in addition to ASA and statins

The proportion of patients that were prescribed OMT are presented in Fig. 2. Comparing the prescription of RAAS-I, BB and MRA at admission and discharge, there was 19, 28 and 18 patients respectively that were initiated on these medications during hospitalization and 18, 8 and 4 patients respectively that discontinued these medications during hospitalization. Hence, there was a significant increase in the prescription of BB and MRA during the hospitalization (*p* < 0.05) but not in RAAS-I (*p* > 0.05). The prescription rate of OMT was significantly increased during hospitalization (44% at admission and 67% at discharge (*p* = 0.004)). Furthermore, the prescription rate of RAAS-I increased from 82% to 96% at 2 months follow-up although not significant (*p* = 0.054) while the prescription rate of BB and MRA increased from 92% to 100% and 40% to 56% (*p* < 0.01). There was no significant difference in OMT between discharge and 2 months follow-up. After 2 months, there were no further increase of the prescription rate in any medication (Fig. 2A).

Defining target-dose OMT as *≥* 50% of target doses of RAAS-I and BB in combination with ASA and statins, the frequency of target-dose OMT was considerably lower with 14 patients (14%) having target-dose OMT at admission and 22 patients (22%) at discharge (*p* = 0.077) (Fig. 2B). At 2 months, there was a non-significant increase of the frequency of patients with target-dose OMT (31 patients (33%), *p* > 0.05) with no further increased in prescription rate at 1 year and 2 years follow-up.

### Patient characteristics and postoperative complications by prescription of optimal medical therapy

All patient at discharge (*n* = 98) were divided into two groups according to prescription of target-dose OMT (Table 1). The target-dose OMT-group had less emergent or urgent CABG compared to the No target-dose OMT group. Almost all patients had multivessel disease but more patients in the No target-dose OMT group had left main disease. No significant differences were observed in age, sex, preoperative renal function, left ventricular function or in medical history (*p* > 0.05).

### Clinical outcomes after surgery and impact of optimal medical therapy

Since 44 patients (43%) had no follow-up echocardiographic exam, these data were highly unreliable due to missing values in almost half of all patients. Hence, no further analysis on echocardiographic data was possible to conduct.

Clinical outcomes were analysed by OMT defined as prescription of RAAS-I, BB, ASA and statins at discharge regardless of target dose. The targe-dose of OMT was not considered in the outcome analysis since many patients increased the prescribed doses during follow-up, which is seen in Fig. 2B. In the OMT group (*n* = 66) 12 patients (18%) died and 12 patients (18%) were hospitalized for heart failure and in the non-OMT group (*n* = 32) 10 patients (31%) died and 13 patients (41%) were hospitalized for heart failure (*p* = 0.146 and *p* = 0.017 respectively). The cause of death were cardiovascular causes for all patient who deceased during hospitalization. For the patient who deceased post-discharge, in total 15 patients died from cardiovascular causes and 4 patients died from other causes. Post-discharge, 7 patients (11%) in the OMT group died from cardiovascular causes and 8 patients (25%) in the non-OMT group died from cardiovascular causes (*p* > 0.05). The cause of death was missing in 7 patients. At 30 days post-surgery, there were two events in the OMT-group and eight events in the No-OMT-group (*p* < 0.002).

**Fig. 3:**
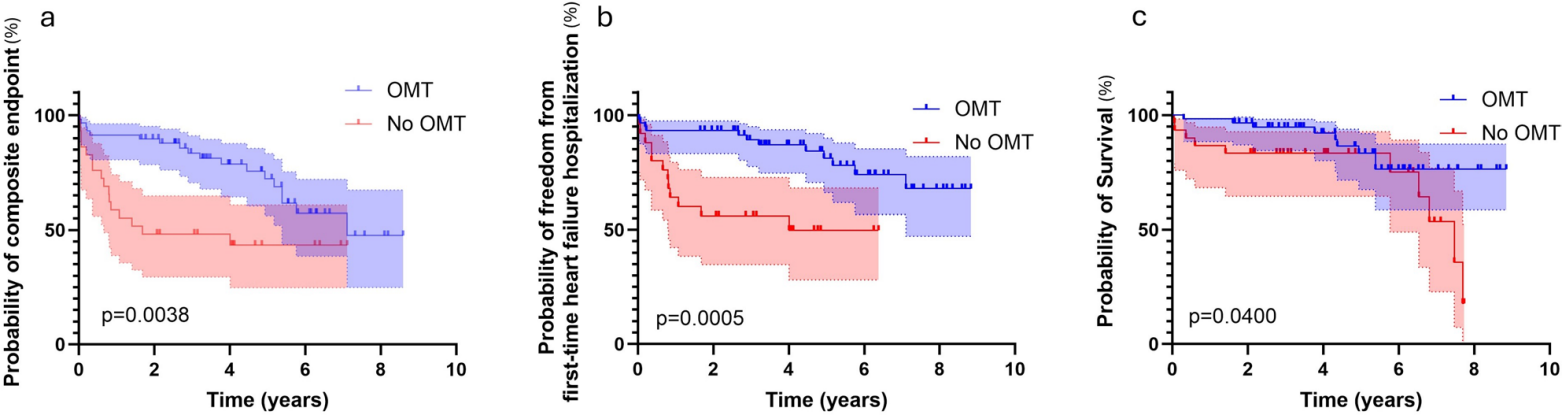
(**a**) Rates of the combined endpoint of cumulative survival and freedom from first-time heart failure rehospitalization by OMT and no-OMT at discharge. (**b**) Rates of freedom from first-time heart failure rehospitalization by OMT and no-OMT at discharge. (**c**) Cumulative survival by OMT and no-OMT at discharge

Patients with OMT at discharge had a significant longer median time to the composite endpoint of first-time hospitalization and all-cause mortality compared to the No-OMT group (1.7 versus 7.1 years, log rank *p* = 0.0038) (Fig. 3A). When analysing the endpoints separately, patients with OMT had a lower risk of first-time heart failure hospitalization as well as a better survival compared to the non-OMT group (log rank *p* < 0.05) (Fig. 3B and C).

The unadjusted univariate analysis showed a reduction in the composite endpoint for patients with higher percentage of target dose RAAS-I, statins, and ASA as well as for OMT (Table 2). Adjusting for clinical important variables that were considering to possible affect the outcome, there was remaining reduction in the composite endpoint for patients with ASA and with higher percentage of target dose RAAS-I. For patients on OMT at discharge, there were an adjusted risk reduction of 75% for the risk of heart failure rehospitalization and all-cause mortality after surgery. Having OMT at discharge was associated with a lower adjusted risk of first-time hospitalization (adjusted hazard ratio (aHR) 0.22 (95% Confidence interval (CI) 0.08–0.57), *p* = 0.002) but not all-cause mortality (aHR 0.51 (95% CI 0.21–1.25), *p* = 0.14). Five patients were excluded due to missing values.

**Table 2:** Prescription of optimal medical therapy at discharge and risk for the composite endpoint

Medical treatment at discharge	HR (95% CI)	*p*	aHR*	*p*-value
RAAS inhibitor (% of target dose)	0.31 (0.11–0.87)	0.03	0.14 (0.03–0.67)	0.012
Beta-blocker (% of target dose)	0.18 (0.09–1.59)	0.18	1.12 (0.25–4.97)	0.884
MRA	0.90 (0.45–1.80)	0.764	1.70 (0.59–4.94)	0.327
Statins	0.26 (0.13–0.53)	< 0.001	0.74 (0.19–2.89)	0.667
ASA	0.28 (0.13–0.62)	0.002	0.05 (0.01–0.32)	0.001
OMT	0.31 (0.17–0.58)	< 0.001	0.25 (0.11–0.58)	< 0.001

*ASA* Acetylsalicylic Acid, *RAAS* renin–angiotensin–aldosterone system inhibitor, *MRA* mineralocorticoid receptor antagonist, *OMT* optimal medical therapy, *HR *Hazard ratio, *aHR* adjusted Hazard ratio, *CI* Confidence interval

*adjusted hazard ratio. OMT was defined as prescription of RAAS-I and BB in addition to statins and ASA at discharge

## Discussion

In this study, we analysed the association between the prescription rate of OMT and the risk of rehospitalization for heart failure and all-cause mortality in a “real-world” population of patients that underwent CABG with a preoperative LVEF *≤* 40%. The prescription rate of OMT increased until the 2 months follow-up with no further increases in dose at 1 year and 2 years follow-up. A considerable number of patients discontinued RAAS-I during hospitalization. Patients that were prescribed OMT at discharge had better outcomes in terms of a reduced risk of hospitalization for heart failure and all-cause death.

About one in three patients that underwent CABG had a preoperative LVEF *≤* 40%, which is in line with results the general CABG-population in Sweden [12]. The proportion of women was comparable to the STICH-trial (13% versus 12%) but lower than the proportion of women undergoing CABG in Sweden [3, 12]. Patients in this study had a higher mean age and a considerable higher number of patients in NYHA class III-IV compared to the STICH-trial, which is often the case of “real-world” observational data compared with controlled randomized trials. However, the proportion of comorbidities was similar compared to the STICH-trial.

We included all patients with a preoperative echocardiography showing a LVEF of 40% or under but only about 70% of the patients had a previous heart failure diagnosis. Since previous studies has showed that up to about one in three develops heart failure after a previous myocardial infarction, the patients without a heart failure diagnosis might been undiagnosed [13]. The predominance of urgent CABG may be an explanation to the low number of OMT at admission. Nevertheless, heart failure medication should be prescribed within the first days after a myocardial infarction complicated by decreased LVEF, including administration of RAAS-Is within the first 24 h after a myocardial infarction, BBs after the first initial 24 h and MRAs within a week [13–16].

The proportion of patients on OMT at admission was higher in our study compared to a post-hoc analysis from the SYNTAX-trial but in line with results from the post-hoc study of STICH-trial [17, 18]. A considerable number of patients in our study was initiated on RAAS-I, BB and MRA during hospitalization but equal number of patients that was initiated on RAAS-I discontinued the treatment. An absolute majority of all patients were prescribed RAAS-I at two months post-surgery. Contraindication in terms of hyperkalaemia might explain the high number of discontinuations of RAAS-I during hospitalisation [10].

Betablockers were the most common medication to initiate during hospitalization. BB are routinely administered to all CABG patients without contraindications since it has a Class IA recommendation in international guidelines, compared with RAAS-I which is not recommended in general but in patients with left ventricular dysfunction, diabetes mellitus and chronic kidney disease [19, 20].

The number of patients with MRA was low at admission but increased during hospitalization and follow-up. Before the updated ESC guidelines 2021, MRA was recommended as a second line treatment in patients with symptomatic heart failure in New York Heart Association (NYHA) class II-IV and LVEF < 35% [10]. Therefore, it is difficult to evaluate the optimal prescription rate. Considering the strong recommendation (Class IA) of statins post-CABG, it was striking that 16% of all patients was discharged without the treatment. Noteworthy, the prescription rate had increased to 97% at the 1-year follow-up. 11% had no ASA at discharge, but no data on other antiplatelet treatment were not available. The prescription rate of ASA declined during follow up, probably due to initiation of oral anticoagulants (OAC) as one in five patients had atrial fibrillation at discharge. The routine in the hospital of this study is to prescribe both ASA and OAC in patients with indications for OAC treatment but the recommended duration of ASA treatment varies, which might reflect a general lack of recommendation on this matter [21].

Another aim of the study was to evaluate the intensity of OMT. Patients with LVEF *≤* 40% should be up titrated on maximal tolerated dose after initiation of OMT [6, 10, 22]. There was an association between increased percentage of target dose RAAS-I and reduced risk of the composite outcome in both in the univariate and the fully adjusted model. No association between target dose of BB and risk of the composite outcome was seen, which could be due to lack of power since treatment with BB has proven survival benefits in patients with heart failure [10]. However, in a previous SWEDEHEART-registry study on secondary prevention after CABG, there was no survival benefits of BB in the fully adjusted model or in the subgroups analysis of patients with LVEF < 50% [12]. Statins were associated with a lower risk of the composite endpoint in the univariate analysis, but not in the adjusted model, indicating that patients not being prescribed statins has other risk factors as well that were associated with higher risks. Prescription of ASA was association of lower risk of the composite outcomes in the adjusted model, but we speculate that it is possible that patients without ASA had other antiplatelet treatment and that these patients may suffer other comorbidities associated with a worse prognosis.

The OMT group had in median five years longer time to the composite endpoint compared to the non-OMT group which mainly was driven by an increased risk the first-time hospitalization for heart failure during the first 2 years post-surgery in the non-OMT group. OMT discharge was associated with a lower adjusted risk of the composite outcome. When analysing the endpoints separately, OMT at discharge was associated with a lower adjusted risk of first-time hospitalization for heart failure but not all-cause mortality. The largest differences in outcome favouring OMT at discharge was seen within the first-year post-surgery. The post-hoc analysis of the STICH-trial also demonstrated a positive effect on outcomes in the OMT-group already after 4 months [18]. However, the risk-benefit ratio and timing of early initiation of OMT after CABG needs to be further evaluated.

### Strengths and limitations

A limitation of this study is that the outcome analysis only included the prescribed medical therapy at discharge, thus, the medical therapy could have changed during the follow-up time. In addition, no data on medication compliance were available. Further, the design of the study as a small, retrospective, single-centre study means that there is a selection bias, a risk for residual confounders in the outcome analysis and a limitation of the generalisability. The small number of included patients may cause an increased risk of a type two error. At last, there is a possibility of reverse causality.

The strength of this study is that the results relay on “real-world” data instead of the controlled setting of a RCT. There are to our knowledge sparse previous publications on the treatment pattern and outcome of heart failure specific therapy in patients with reduced LVEF undergoing CABG.

## Conclusions

In this single-center observational study, patients with prescribed OMT at discharge had a better outcome with lower incidence of first-time hospitalization for heart failure and all-cause death, mainly during the first two years post-surgery. This study suggests an early initiation of OMT in patients undergoing CABG with reduced LVEF. Further studies are needed to evaluate the risk-benefit ratio of early initiation and up-titration of OMT in patients with reduced LVEF undergoing CABG.

## Data Availability

The data underlying this article cannot be shared publicly due to ethical restrictions, as data contain potentially identifying or sensitive patient information, which is imposed by Ethical Review Board. The datasets used and/or analysed during the current study are available from the corresponding author on reasonable request.

## Abbreviations

CABG: Coronary artery bypass graft
RAAS-I: Renin-angiotensin-aldosterone system inhibitor
BB: Beta-blocker
MRA: Mineralocorticoid receptor antagonist
ASA: Acetylsalicylic acid
OMT: Optimal medical therapy
ACE-I: Angiotensin-converting-enzyme inhibitor
ARB: Angiotensin II receptor blocker
SD: Standard deviation
HR: Hazard ratio
aHR: Adjusted hazard ratio
